# Supplementary material for: Disrupting abnormal neuronal oscillations with adaptive delayed feedback control
Source: eLife. 2024 Mar 7;13:e89151. doi: 10.7554/eLife.89151 (PMC10987087; doi:10.7554/eLife.89151)
Supplement: Supplementary file 1. — We used repeated measures one-way ANOVA with multiple comparisons. [file elife-89151-supp1.docx]

**Supplementary File 1 (Table S1).** Details of the statistical tests used in Figure 4 to compare the modulation results of the different stimulation protocols in controllable networks. We used repeated measures one-way ANOVA with multiple comparisons.

|  | | **Oscillation Intensity** | | **Synchrony** | | **Firing Rate** | |
| --- | --- | --- | --- | --- | --- | --- | --- |
| **Comparison** | **n** | **Mean Diff.** | **p values** | **Mean Diff.** | **p values** | **Mean Diff.** | **p values** |
| Control vs aDFC | 8 | **5.290** | **0.0048** | **0.1266** | **0.0454** | **-0.1600** | **0.0082** |
| Control vs DFC | 8 | **-3.242** | **0.0164** | 0.1075 | 0.1313 | -0.3332 | 0.2651 |
| Control vs Poisson | 8 | **4.835** | **0.0039** | 0.07756 | 0.1817 | -0.2281 | 0.2071 |
| aDFC vs DFC | 8 | **-8.532** | **0.0012** | -0.01904 | 0.5039 | -0.1732 | 0.6420 |
| aDFC vs Poisson | 8 | -0.4550 | 0.9098 | **-0.04899** | **0.0142** | -0.06806 | 0.8459 |
| DFC vs Poisson | 8 | **8.077** | **0.0029** | -0.02995 | 0.4456 | 0.1051 | 0.4577 |
