# Supplementary material for: Disrupting abnormal neuronal oscillations with adaptive delayed feedback control
Source: eLife. 2024 Mar 7;13:e89151. doi: 10.7554/eLife.89151 (PMC10987087; doi:10.7554/eLife.89151)
Supplement: Supplementary file 2. — We used repeated measures one-way ANOVA with multiple comparisons. [file elife-89151-supp2.docx]

**Supplementary File 2 (Table S2).** Details of the statistical tests used in Figure 7 to compare percentage of time spent in asynchronous state for each stimulation protocol in a multistable network. We used repeated measures one-way ANOVA with multiple comparisons.

|  | **aDFC** | | | **DFC** | | | **Poisson** | | |
| --- | --- | --- | --- | --- | --- | --- | --- | --- | --- |
| **Comparison** | **n** | **Mean Diff.** | **p values** | **n** | **Mean Diff.** | **p values** | **n** | **Mean Diff.** | **p values** |
| OFF_pre_ - ON | 11 | **-0.3072** | **<0.0001** | 7 | -0.02274 | 0.771 | 6 | -0.1208 | 0.3023 |
| ON - OFF_pos_ | 11 | **0.3052** | **<0.0001** | 7 | -0.07382 | 0.3143 | 6 | 0.07317 | 0.9952 |
| OFF_pre_ - OFF_pos_ | 11 | -0.002 | 0.9989 | 7 | -0.09656 | 0.1706 | 6 | -0.1135 | 0.3444 |
