# Supplementary material for: Disrupting abnormal neuronal oscillations with adaptive delayed feedback control
Source: eLife. 2024 Mar 7;13:e89151. doi: 10.7554/eLife.89151 (PMC10987087; doi:10.7554/eLife.89151)
Supplement: Supplementary file 3. — We used repeated measures one-way ANOVA with multiple comparisons. [file elife-89151-supp3.docx]

**Supplementary File 3 (Table S3).** Details of the statistical tests used in Figure 3-figure supplement 2 to compare fraction of spontaneous spikes during stimulation for the three stimulation protocols. We used repeated measures one-way ANOVA with multiple comparisons.

| **Comparison** | **n** | **Mean Diff.** | **p values** |
| --- | --- | --- | --- |
| aDFC vs DFC | 8 | **-0.2372** | **0.0089** |
| aDFC vs Poisson | 8 | **-0.1909** | **0.0181** |
| DFC vs Poisson | 8 | 0.04628 | 0.4459 |
